# Geothermal and structural features of La Palma island (Canary Islands) imaged by ambient noise tomography

**DOI:** 10.1038/s41598-023-39910-z

**Published:** 2023-08-09

**Authors:** Iván Cabrera-Pérez, Jean Soubestre, Luca D’Auria, José Barrancos, Alba Martín-Lorenzo, David Martínez van Dorth, Germán D. Padilla, Monika Przeor, Nemesio M. Pérez

**Affiliations:** 1 Instituto Volcanológico de Canarias (INVOLCAN), 38600 Granadilla de Abona, Tenerife, Canary Islands, Spain; 2 Univ. Grenoble Alpes, Univ. Savoie Mont Blanc, CNRS, IRD, Univ. Gustave Eiffel, ISTerre, 38000 Grenoble, France; 3 Icelandic Meteorological Office, Reykjavik, Iceland; 4 Instituto Tecnológico y de Energías Renovables (ITER), 38600 Granadilla de Abona, Tenerife, Canary Islands, Spain

**Keywords:** Solid Earth sciences, Geophysics, Seismology, Volcanology

## Abstract

La Palma island is located in the NW of the Canary Islands and is one of the most volcanically active of the archipelago, therefore the existence of geothermal resources on the island is highly probable. The main objective of this work is to detect velocity anomalies potentially related to active geothermal reservoirs on La Palma island, by achieving a high-resolution seismic velocity model of the first few kilometres of the crust using Ambient Noise Tomography (ANT). The obtained ANT model is merged with a recent local earthquake tomography model. Our findings reveal two high-velocity zones in the island’s northern and southern parts, that could be related to a plutonic intrusion and old oceanic crust materials. Conversely, four low-velocity zones are imaged in the southern part of the island. Two of them can be related to hydrothermal alteration zones located beneath the Cumbre Vieja volcanic complex. This hypothesis is reinforced by comparing the S-wave velocity model with the seismicity recorded during the pre-eruptive phase of the 2021 Tajogaite eruption, which revealed an aseismic volume coinciding with these low-velocity zones. Another low-velocity zone is observed in the southern part of the island, which we interpret as highly fractured rocks which could favour the ascent of hot fluids. A last low-velocity zone is observed in the central part of the island and associated with loose deposits generated by the Aridane valley mega landslide.

## Introduction

**Figure 1:**
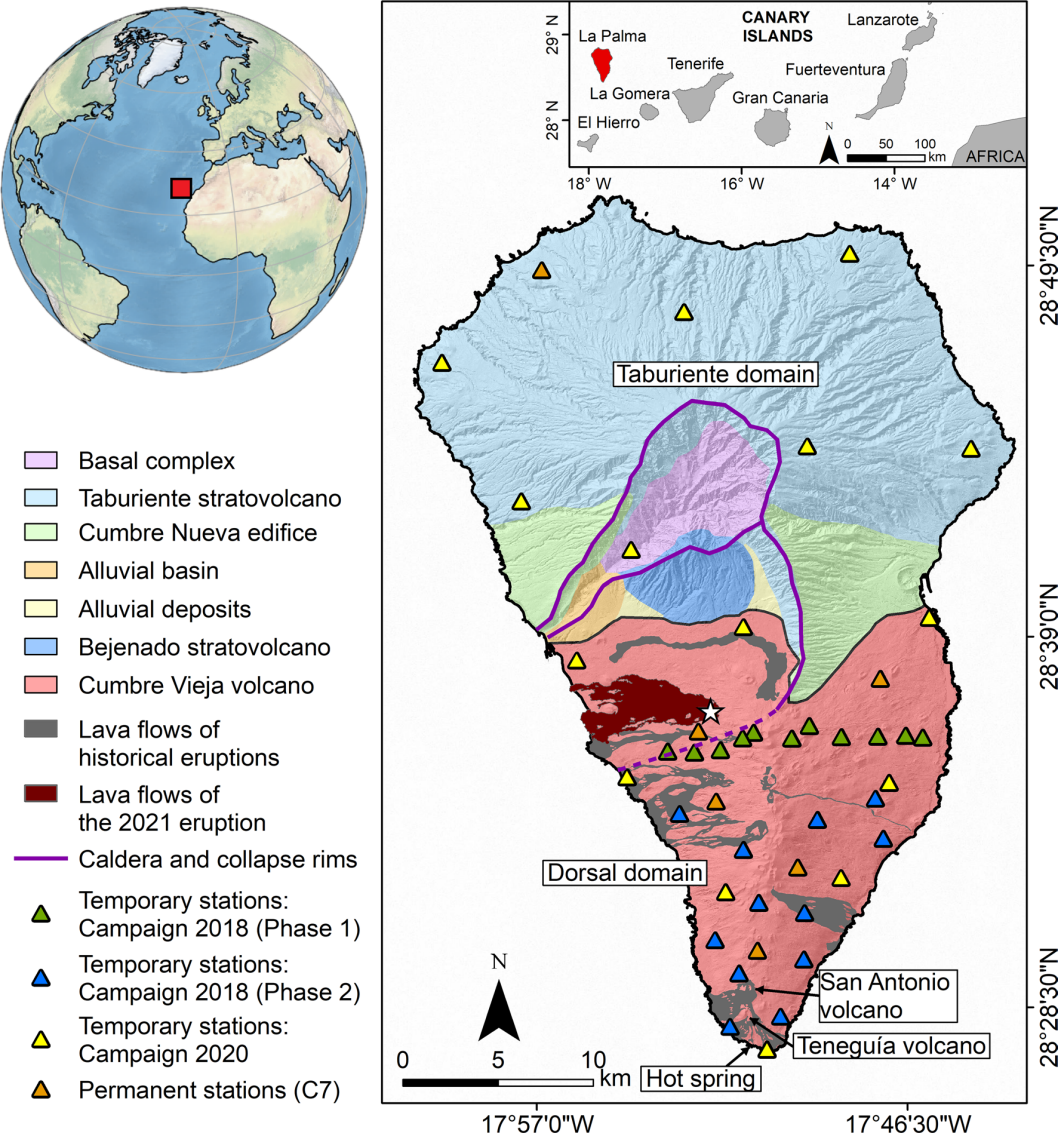
Geological map of La Palma island (modified from Padrón et al.^46^). The white star represents the location of the Tajogaite eruption, the last eruption which took place on the island between September 19th and December 13th, 2021. The triangles represent the location of temporal and permanent seismic stations, with colors corresponding to different phases as described in the caption.

The Canary Islands archipelago comprises seven islands located close to the northwest coast of Africa, between latitudes 27$^{\circ }$38’N and 29$^{\circ }$25’N, and longitudes 13$^{\circ }$20’W and 18$^{\circ }$90’W (Fig. 1). All these islands have a volcanic origin. Volcanism in the Canary Islands began during the Oligocene and is still active^1^. La Palma island is located in the western part of the archipelago and is one of the youngest islands. The island has an elongated shape following a North-South direction with a maximum height of 2426 m a.s.l. (above the sea level). Two very well differentiated geological domains can be distinguished on La Palma island: the Taburiente Domain, which is the oldest domain located in the northern part of the island, and the Cumbre Vieja volcanic complex, which is constituted by the most recent volcanic materials in the southern part of the island (Fig. 1). The island’s formation began with submarine lava emissions 4 My ago. The first subaerial material was emitted 1.77 My ago, forming the Taburiente stratovolcano. Between 0.77 and 0.56 My, the Cumbre Nueva volcanic complex formed to the South of the Taburiente stratovolcano. During this period, a landslide occurred on the western flank of Cumbre Nueva, creating the large Aridane valley in the central part of the island^2^. Between 0.56 and 0.49 My, the Bejenado stratovolcano was formed on top of the Cumbre Nueva edifice. Finally, the Cumbre Vieja volcanic complex started its formation 0.12 My ago. It is currently the only volcanically active zone of the island, where all the historical eruptions took place.

The last volcanic eruption on La Palma island started on September 19th, 2021, and lasted approximately three months up to December 13th. This eruption, named Tajogaite eruption, resulted in significant social, economic and scientific impacts. Pre-eruptive unrest started on September 11st, 2021, eight days before the eruption^3^. During this unrest, the seismicity quickly migrated from 10 km depth to the surface, following the ascending path of the magma. Finally, a fissural eruption began on September 19th at about 14:00 UTC, with a dominantly strombolian activity and episodic phreatomagmatic pulses^4,5^. This latest eruption has reminded the potential of this island to host geothermal resources. By the way, the interest for geothermal exploration had recently been renewed in the Canary Islands, as testified by several geophysical and geochemical studies realized on Gran Canaria island^6–8^, Tenerife island^7,9–12^ and La Palma island^13^.

The present study aims at determining the geological structure of La Palma island through seismic ambient noise tomography (ANT), focusing on velocity anomalies possibly related to geothermal reservoirs. The ANT has proven to be an efficient method to image structures at different scales e.g.,^14–25^. The first ANT dedicated to geothermal exploration was realized by Yang et al.^26^ at the Coso geothermal field (California) and revealed the existence of shallow low-velocity zones related to geothermal alteration. Other studies of ANT inferred the presence of temperature anomalies related to deep hydrothermal circulation e.g.,^8,27,28^, water reservoirs e.g.,^29^, new geothermal reservoirs e.g.,^30^, pockets of partial melt e.g.,^31^ or deep heat sources e.g.,^32^. These studies evidenced the potential of ANT as a complementary geophysical method for geothermal exploration.

Data acquisition was realized between 2018 and 2020, by deploying 38 broadband seismic stations during different campaigns (Fig. 1). The methodology used for the ANT is detailed in the "Methods" section. The ANT first comprises a non-linear multiscale inversion taking the topography into account^33^ to retrieve the 2-D group velocity maps at different periods. Those group velocity maps are then inverted at depth using a transdimensional approach^34^. The obtained 3-D S-wave velocity model of the island is exposed and discussed in the following sections. It is compared with previous geophysical studies^3,13,35^ to determine common features. Furthermore, the ANT model is combined with a recent local earthquake tomography (LET) model^3^, having ANT a higher resolution at shallow depth but a limited penetration depth compensated by the LET.

## Results

**Figure 2:**
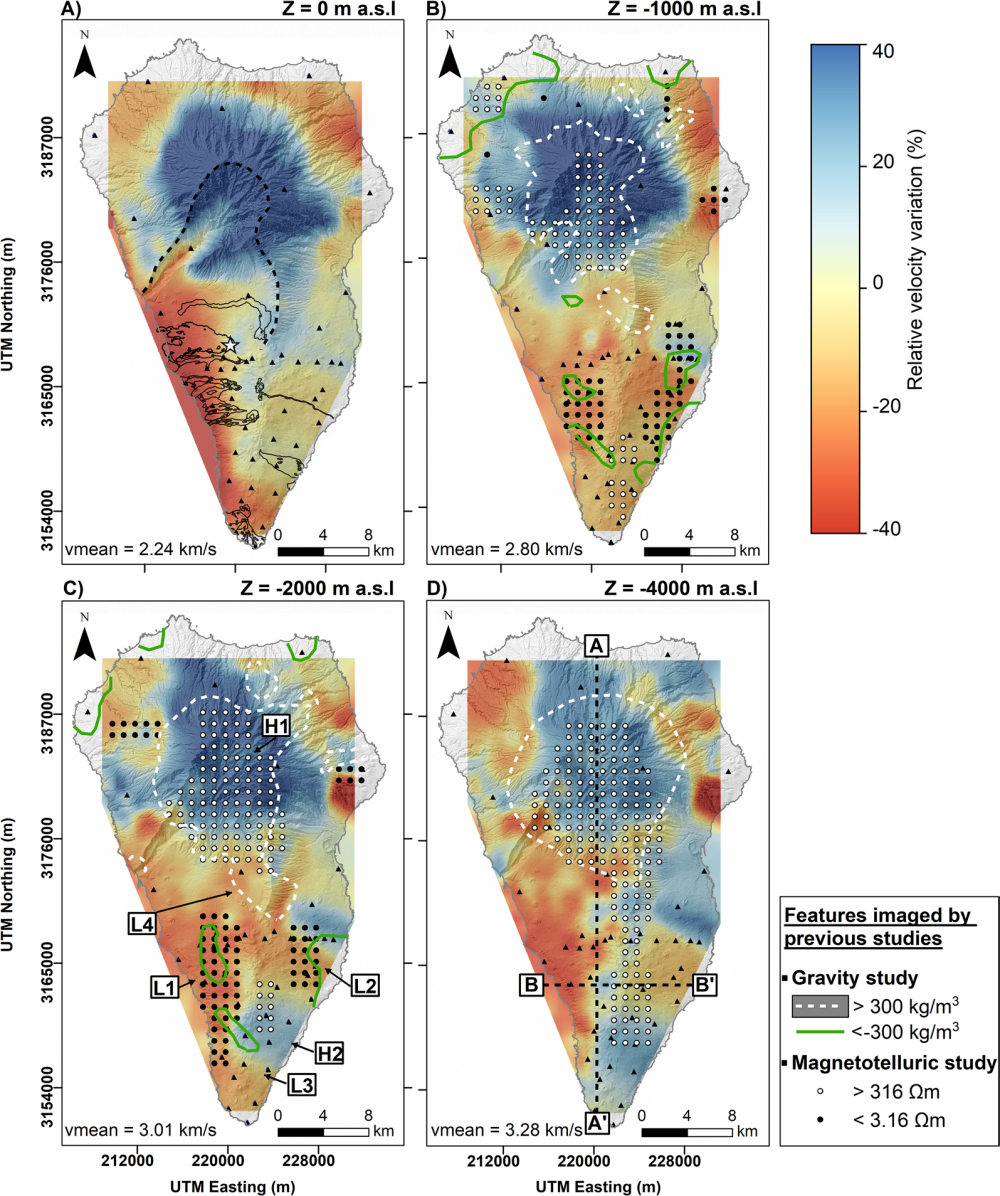
S-wave relative velocity maps at different depths. (**A**) The black lines and white star represent the lava flows of the historical eruptions and the location of the Tajogaite volcano, respectively. The black triangles show the location of seismic stations. The white and green lines in subplots (**B**–**D**) represent the high and low-density anomalies from a previous density study^35^, respectively. The white and black points in subplots (**B**–**D**) represent the high and low resistivity zones from a previous magnetotelluric study^13^, respectively. The different seismic velocity anomalies obtained from this study and discussed in the text are shown in subplot (**C**). Vertical cross-sections corresponding to Figs. 3 and 4 are shown in subplot (**D**).

This section illustrates the results of the ambient noise tomography. Figure 2 shows the obtained maps of S-wave relative velocity variation at different depths. The relative velocity variation at each depth is calculated with respect to the mean velocity at the corresponding depth. The main anomalies observed in the S-wave velocity model are marked in Figure 2C, distinguishing between high (H) and low (L) velocity anomalies. It can be seen on Figure 2 that the island shows very strong velocity variations, reaching 40% in some areas. The mean S-wave velocity is 2.24 km/s in superficial zones (Fig. 2A), while it reaches 3.28 km/s for deeper zones (Fig. 2D).

**Figure 3:**
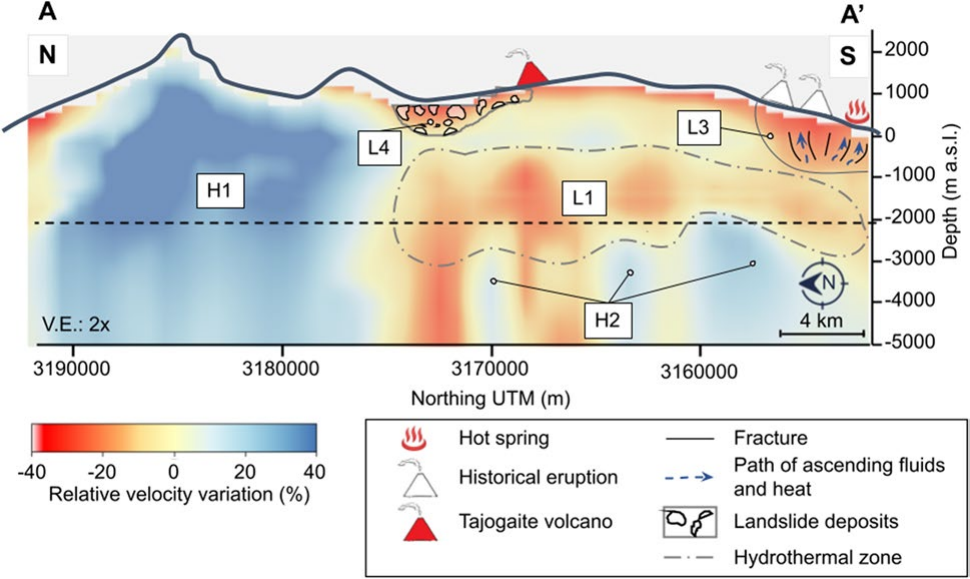
Vertical N–S cross-section (cf. A–A’ in Fig. 2D) of the S-wave relative velocity model. The black dashed line represents the depth at which the model resolution is maximum and below which it starts to decrease. White and red triangles represent the historical and Tajogaite eruption sites, respectively.

**Figure 4:**
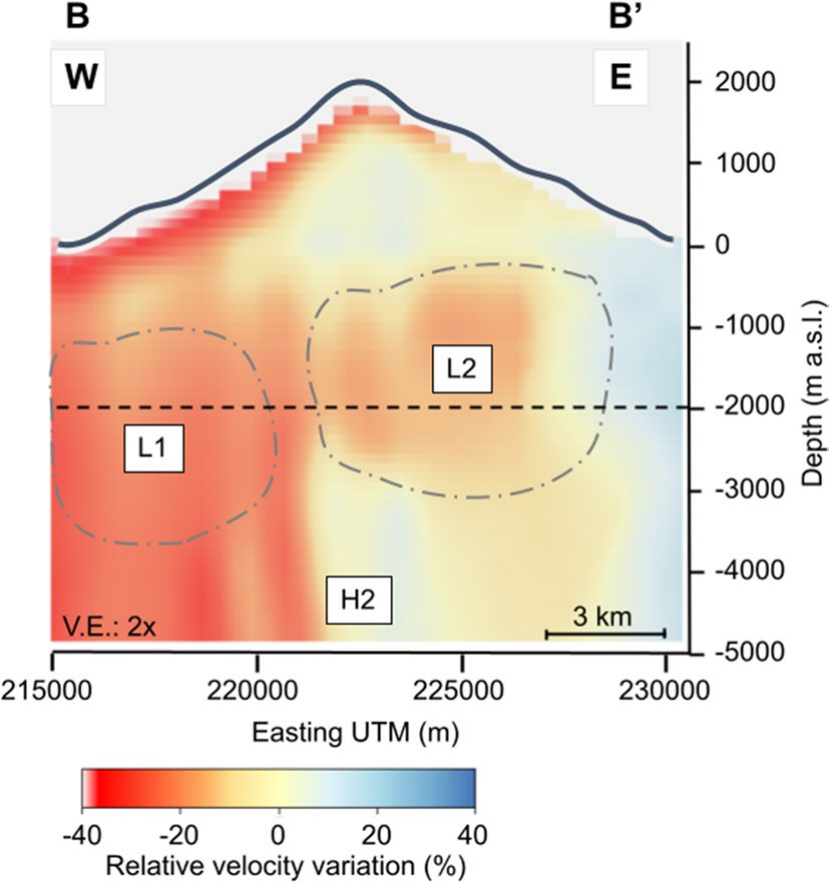
Vertical E-W cross-sections (cf. B–B’ in Figure 2D) of the S-wave relative velocity model. The black dashed line represents the depth at which the model resolution is maximum and below which it starts to decrease.

Figures 3 and 4 show two vertical cross-sections of the 3-D S-wave velocity model, which positions are indicated in Fig. 2D. A high-velocity zone (H1) extends from the surface to a depth of 5000 m b.s.l. (below sea level) in the island’s northern part, with relative velocities that vary between 20% and 40% (Fig. 3). Another high-velocity zone (H2) is present between 2000 m b.s.l. and 5000 m b.s.l. in the island’s southern part (Fig. 3). On the other hand, two low-velocity zones (L1 and L2) can be observed in the southern part of the island, located on the western (L1) and eastern (L2) flanks of the Cumbre Vieja volcanic complex (Fig. 2C). These velocity anomalies are found at a depth ranging from 0 down to 3000 m b.s.l. (Fig. 4) with relative velocity variations ranging between $-20\%$ and $-40\%$. A third low-velocity anomaly (L3) is located in the southern part of the Cumbre Vieja volcanic complex at a depth between 0 and 1000 m b.s.l., with relative velocities of less than $-20\%$ (Fig. 3). Finally, a fourth low-velocity anomaly (L4) can be observed in the central part of the island, with a depth not exceeding the sea level and relative velocity variations of $-20\%$ (Fig. 3). As detailed in the "Methods" section, some resolution tests confirmed the capacity of the used network configuration to image all the previously mentioned anomalies (Figs. S4, S5 and S6 from the supplementary materials).

**Figure 5:**
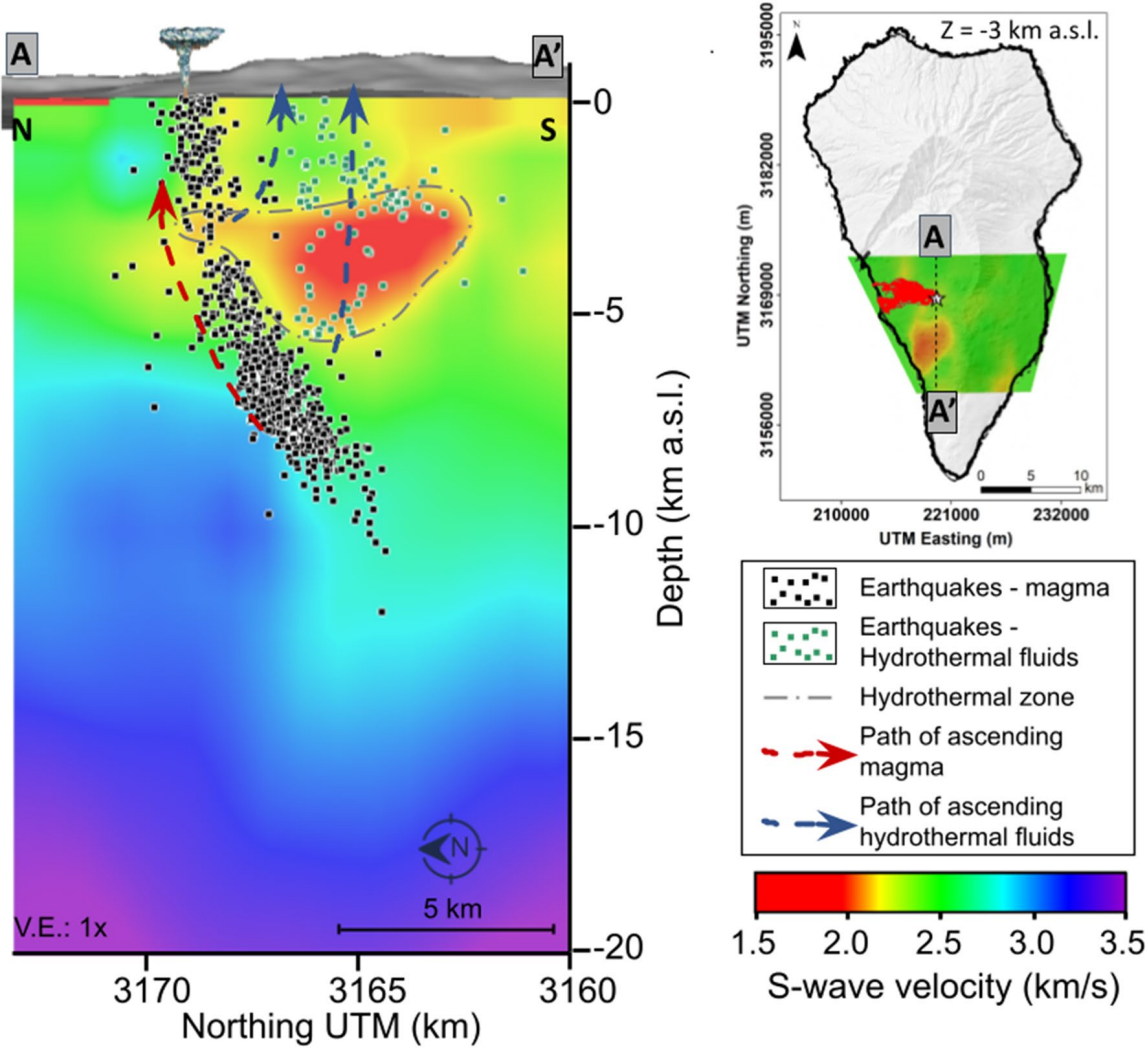
Vertical N–S cross-section of the 3-D unified S-wave velocity model obtained from local earthquake tomography (LET, previous study) and ambient noise tomography (ANT, this study). The black and green dots represent earthquakes related to magma and hydrothermal fluid ascent, respectively.

Figure 5 shows a vertical cross-section of the unified ANT+LET S-wave velocity model together with the hypocenters of the earthquakes recorded during the Tajogaite eruption pre-eruptive unrest (11-19 Sept. 2021). This seismicity was relocated by D’Auria et al.^3^ using a tridimensional velocity model obtained from LET. This allowed determining the path of the ascending magma to the surface. The unified S-wave velocity model (Fig. 5) shows some relevant velocity anomalies. A high-velocity zone is observed below 5 km b.s.l., whose depth increases in the southern part of the island. D’Auria et al.^3^ related this feature to the geometry of the Moho beneath the island. In addition, a low-velocity anomaly is clearly observed at shallow depth ($< 5$ km b.s.l.) and coincides with anomalies detected by other geophysical studies in the same area^13,35^, as discussed in the next section.

## Discussion

The 3-D S-wave velocity model of La Palma island reveals a great complexity of the geological structure with six principal velocity anomalies: two high-velocity anomalies located in the northern and southern parts of the island, and four low-velocity anomalies associated with the Cumbre Vieja volcanic complex in the southern part of the island. The approach we used for the tomographic inversion takes into account the topography of the island. Furthermore, the use of a transdimensional approach for depth inversion does not require establishing an “a priori” parametrization and provides velocity profiles in a Bayesian fashion, which allows a quantitative non-linear estimation of the uncertainties. In the following, we discuss the volcanological and geothermal relevance of such anomalies, making a comparison with the resistivity model of Di Paolo et al.^13^ and the density model of Camacho et al.^35^.

The anomaly H1 is one of the most relevant due to its giant size. It has already been observed by other geophysical studies. Camacho et al.^35^ observed a high-density body in this zone, with values above 300 kg/$m^{3}$. In the same zone, Di Paolo et al.^13^ imaged a high-resistivity anomaly, with peak values exceeding 316 $\Omega m$ (Fig. 2). Considering these studies and our S-wave velocity model, this anomaly is interpreted as linked to the plutonic intrusion related to the ancient volcanism of the island during its basal complex formation (ca. 4.0 to 3.0 Ma)^1,36^.

Another group of significant high-velocity anomalies (H2) coincides with a high-resistivity zone (Figs. 2C and D) with values higher than 316 $\Omega m$ (Di Paolo et al.^13^). We note that the density model of Camacho et al.^35^ doesn’t show any anomaly in this zone. Di Paolo et al.^13^ suggest that this anomaly could be related to the upper part of a shallow intrusive magmatic complex. This hypothesis is compatible with our velocity model. Such intrusive bodies were observed in other ANT studies realized in Snæfellsjökull volcano^37^, Eyjafjallajökull volcano^38^, Misti volcano^25^, and other volcanic and geothermal environments.

From a geothermal point of view, the most relevant features are the low-velocity anomalies L1 and L2 (Fig. 2). Both anomalies coincide with zones of low resistivity and low density. Camacho et al.^35^ associated these anomalies with zones of shallow fractures following the direction of the N-S rift structure of Cumbre Vieja. On the other hand, Di Paolo et al.^13^ interpreted these anomalies as clay alteration caps (illite and illite-smectite). Because of the geophysical characteristics detailed hereafter, we consider that these low-velocity anomalies are related to a hydrothermal alteration zone which could indicate the presence of an active or fossil hydrothermal system in the Cumbre Vieja volcanic complex. Previous studies demonstrated that hydrothermal fluids could decrease the S-wave velocity^39,40^. This hypothesis matches observations from our unified S-wave velocity model and its comparison with the hypocenters of the pre-eruptive seismicity (Fig. 5). The seismicity recorded during the pre-eruptive episode of the Tajogaite eruption indicates a nearly vertical ascent of the magma to the surface (Fig. 5, black dots), which rose through a network of interconnected sills and dykes^41^. However, the seismicity south of the eruption site (Fig. 5, green dots) was associated with the ascent of hydrothermal fluids^3,42^. Cabrera-Pérez et al.^42^ indirectly confirmed the existence of a possible geothermal reservoir in the western flank of the Cumbre Vieja volcanic complex through ambient noise interferometry by computing relative velocity variations (dv/v) during the pre-eruptive phase of Tajogaite eruption. The authors observed a decrease of dv/v in this zone prior to the eruption, which they interpreted as the ascent of hydrothermal fluids exsolved from the magma, which reached the surface only a few days later. Furthermore, recent petrological observations realized by Pankhurst et al.^43^ determined that the magmas emitted during the initial phase of the Tajogaite eruption were more hydrated, which could indicate that this hydrothermal alteration zone is still active, with the current presence of fluids. These fluids would travel faster through zones of high permeability caused by intense fracturing, such as the low-velocity anomaly L1 imaged by our S-wave velocity model. Moreover, the number of earthquakes is considerably reduced in this low-velocity zone (Fig. 5, green dots). This could be a consequence of the presence of hydrothermally altered material characterized by a lower rigidity, which could explain the reduced seismogenic capability of this zone. Conversely, the microseismic activity is higher and more important outside of this zone (Fig. 5, black dots), indicating a more fragile behaviour. Those different geophysical and petrological observations sustain our hypothesis of the existence of an active hydrothermal system beneath the Cumbre Vieja volcanic complex.

Another low-velocity anomaly is L3, located in the southern part of the island (Fig. 2C), beneath the two historical eruptions of San Antonio (1677) and Teneguía (1971) volcanoes (Fig. 3). This zone of low velocity does not coincide with any resistivity or density anomaly observed in the studies of Di Paolo et al.^13^ and Camacho et al.^35^. However, subsidence was detected by InSAR beneath Teneguía volcano by Prieto et al.^44^, which they related to a thermal source. In addition, Padrón et al.^45^ measured an anomaly of diffuse $CO_{2}$ emission ( > 800 $g\ m^{-2} day^{-1}$) in the area of Teneguía volcano prior to the Tajogaite eruption (2001-2013). Moreover, they sampled anomalous temperatures varying between 90$^{\circ }$C and 130$^{\circ }$C at 40 cm depth in this zone. It should be noted that a hot spring (Fuente Santa) is present in the southern part of the Teneguía volcano (Figs. 1 and 3 ), where water temperatures reaching 40$^{\circ }$C and concentration levels of $\text {HCO}_{3}^{-}$ and $\text {SO}_{4}^{2-}$ exceeding 2000 mg/L were measured^46^, which could indicate the circulation of underground water through high-temperature rocks. Our hypothesis to explain the low-velocity anomaly L3 is that high-temperature rocks and a series of fractures through which hydrothermal fluids and gases are rising to the surface are present under the Teneguía and San Antonio volcanoes, which would explain the observed geochemical anomalies^45,46^ and subsidence^44^.

Finally, the low-velocity anomaly L4 is located in a valley zone of the island’s central part (Fig. 2C). This valley was produced by at least two destructive episodes related to huge landslides that formed the arc of Cumbre Nueva and the Taburiente caldera, partially destroying the existing volcanic edifices^2^. This anomaly could be related to landslide deposits mostly composed of conglomerate materials.

Considering the previous geophysical models^3,13,35^ and the S-wave velocity model obtained from ANT in this study, we argue for the highest geothermal potential of La Palma island to be located on both the western and eastern flanks of the active Cumbre Vieja volcanic complex at a depth of 2000 m b.s.l., approximately. In addition, the southern part of this volcanic system also seems to host some shallow geothermal resources, as evidenced by this study and additional geophysical^44^ and geochemical^45,46^ observations.

## Conclusions

A 3-D S-wave velocity model of La Palma island was obtained by unifying results from a new ANT model obtained in this study and a LET model obtained by D’Auria et al.^3^. We applied ANT on data recorded by 38 broadband seismic stations to extract the dispersion curves of all the station pairs. Subsequently, we obtained the 2-D group velocity maps through a non-linear multiscale inversion taking the topography into account^33^. Finally, we derived some S-wave 1-D profiles using a Bayesian tridimensional inversion. The final 3-D S-wave velocity model shows the presence of two high-velocity zones (H1 and H2) and four low-velocity zones (L1, L2, L3 and L4).

The high-velocity anomalies H1 and H2 are interpreted as related to a plutonic intrusion related to the island’s ancient volcanism and more recent solidified intrusive dyke complexes, respectively. From the point of view of geothermal exploration, the most interesting imaged features are the low-velocity anomalies L1, L2, and L3. The low-velocity zones L1 and L2 are interpreted as hydrothermal alteration zones associated with the presence of an active or fossil hydrothermal system in the Cumbre Vieja volcanic complex. Velocity variations estimated before the Tajogaite eruption seem to favour the hypothesis of an active hydrothermal reservoir. The anomaly L3 is interpreted as associated with fractured rocks favouring the ascent of hot fluids toward the surface in the island’s southern part. This hypothesis could also explain the geochemical and geophysical anomalies observed in previous studies^44–46^. Finally, the low-velocity anomaly L4 could be related to landslide deposits produced during destructive episodes of the island’s geological history.

It would be necessary to carry out more detailed geophysical and geochemical exploration surveys at the scale of the Cumbre Vieja volcanic complex in order to further advance in the characterization of the geothermal potential of La Palma island. This is especially true in the southern part of the island, where S-wave low-velocity anomalies are shallower. Furthermore, it would be necessary to apply clustering and machine learning techniques to realize a quantitative comparison of the resistivity, density and S-wave velocity models, in order to better interpret the geological context.

## Methods

### Data acquisition

In 2018 we installed 23 seismic stations in the southern part of the island, in two phases of one month each, focusing on the Cumbre Vieja volcano. In 2020 we installed 15 stations distributed throughout the whole island which were recording during two months. The goal was to characterize the zones that were not sampled during the first two campaigns. Considering the distribution of the seismic stations, the highest density of stations and ray path anisotropy is in the Cumbre Vieja volcano (Fig. S1 in the supplementary materials), which is the most active part of the island from a volcanological point of view. Furthermore, we used six permanent stations operated by Instituto Volcanológico de Canarias for volcano monitoring (Fig. 1).

### Ambient noise data processing

In order to realize the ambient noise data processing we pre-processed the data, cross-correlated all the station pairs and extracted the dispersion curves^14^, as detailed hereafter. We used an automatic network-based method^47,48^ to remove time windows containing earthquakes (Fig. S2 in the supplementary materials). Subsequently, a bandpass filter was applied in the 0.01–5.00Hz frequency range and a standard ambient noise pre-processing composed of temporal one-bit normalization and spectral whitening was applied to the remaining data to reduce its non-stationarity^49^.

Afterwards, the cross-correlations of pre-processed data were computed for all station pairs on five-minute-long windows and stacked over one month for the 2018 campaigns and two months for the 2020 campaign. We performed this analysis on vertical-vertical components for 578 station pairs. The obtained cross-correlations appear in Figure S3.A, evidencing coherent wavetrains of dispersive Rayleigh waves. In order to verify that the noise sources distribution was close to isotropy, which is a fundamental assumption when doing ANT^50,51^, we calculated the amplitude ratio of the causal and acausal parts of cross-correlations as a function of the azimuthal distribution of station pairs (Fig. S3B). Figure S3B shows an excellent azimuthal distribution for all orientations of station pairs. Furthermore, it seems that there is no specific dominating noise source, as the amplitude ratio is mostly close to 1 for all the station pairs (Fig. S3B).

Then, the Rayleigh wave group velocity dispersion curves were determined through frequency-time analysis (FTAN)^52^. We extracted 415 dispersion curves shown in Figure S3.C. The red curve represents the mean dispersion curve with its standard deviation at each period (blue line). Figure S3.D shows the number of measurements as a function of the period. We limit our analysis to reasonably covered period ranges with at least 50 measurements, restricting us to periods between 0.35 s and 3.2 s for the tomographic inversion.

### 2-D group velocity maps

We applied a non-linear multiscale inversion taking the topography into account^33^ to obtain the 2-D group velocity maps at different periods. The starting model consists of a homogeneous velocity model. The model parametrization is refined at subsequent non-linear inversion steps by adding control nodes over a regular grid. In other words, at each step, we refine the model by increasing the scale, which means increasing the number of parameters used to define the model. The result of each scale is used as a starting model for the following inversion scale. This inversion method was applied satisfactorily in different studies of volcanic areas^8,25^.

We performed different tests on synthetic models to determine the spatial resolution of the tomographic images. First, checkerboard tests (Fig. S4 in the supplementary materials) and a test on a synthetic model composed of a pattern of low and high-velocity diamond-shaped anomalies (Fig. S5 in the supplementary materials) were performed. We used three different linear sizes for both tests, namely 0.05$^{\circ }$ x 0.05$^{\circ }$, 0.1$^{\circ } \times 0.1^{\circ }$ and 0.2$^{\circ }$ x 0.2$^{\circ }$, corresponding approximately to sizes of 5.5 km x 5.5 km, 11.1 km x 11.1 km and 22.2 km x 22.2 km, respectively, with a maximum velocity of 2.1 km/s and a minimum of 1.9 km/s (Figs. S4 and S5 in the supplementary materials). Both models with a resolution of 0.1$^{\circ }$ x 0.1$^{\circ }$ and 0.2$^{\circ }$ x 0.2$^{\circ }$ were correctly retrieved (Figs. S4.D, S4.F, S5.D and S5.F in the supplementary materials). Conversely, the checkerboard test with a resolution of 0.05$^{\circ }$ x 0.05$^{\circ }$ was not correctly retrieved in the island’s northern part (Figs. S4.B and S5.B) due to a lower ray path density in this zone (Fig. S1), but it was correctly retrieved in the southern part of the island where the ray path density is higher (Figs. S4B, S5B and S1 in the supplementary materials). Furthermore, a resolution test was realized on a synthetic model composed of anomalies similar to anomalies H1, L1, and L2 imaged from real data (Fig. S6A in the supplementary materials). Figure S6.B shows that the three anomalies were correctly retrieved.

Figure S7 of the supplementary materials shows the results of the 2-D mapping of the Rayleigh wave group velocity obtained using a checkerboard test at different scales of 4, 5 and 6. Maps of scales 4 and 5 are pretty similar and correctly retrieve both the geometry and velocity. Conversely, the map of scale 6 shows artefacts, and the velocity pattern cannot be retrieved correctly. Therefore, we limit our inversion process on real data to a scale of 5.

Figure S8 shows four Rayleigh wave 2-D group velocity maps obtained from real data for periods between T = 1.2 s and T = 3.0 s. The variance reduction(VarRed) and mean velocity are indicated on each panel of Figure S8. For all the considered periods, the VarRed is higher than 50%. On the other hand, the mean velocity increases with the period, from 1.22 km/s to 1.81 km/s, consistently with the global trend of the average dispersion curve shown in Figure S3.C.

Regarding the spatial patterns in the 2-D group velocity maps (Fig. S8 in the supplementary materials), we observe important velocity contrasts at different periods. At short period corresponding to shallow depth, the T = 1.2 s map does not show significant anomalies, predominating the low-velocity zone in the great majority of the island with a mean velocity of 1.2 km/s. At higher period, the T = 2.00 s map shows a velocity increase in the island’s northern part, which increases again at higher periods. Between the periods of T = 2.5 s and T = 3.0 s, we can observe the presence of two low-velocity zones on both flanks of the Cumbre Vieja volcanic complex, with a velocity lower than 1.25 km/s.

### Depth inversion

The last step in the inversion process involves inverting the group velocity maps to obtain S-wave 1-D profiles in depth. We extracted a dispersion curve corresponding to each of the 347 control points of the 2-D group velocity maps to perform the inversion using a transdimensional approach^34^. This approach allows obtaining an “a posteriori” probability distribution of the seismic velocities, largely independent of a specific parametrization, namely the number of layers. The transdimensional approach includes the parametrization itself among the inverse problem parameters^53^. In this work, we explored models having a number of uniform horizontal layers ranging between one and five. The calculation of the forward model used for the computation of dispersion curves was performed using a modified Thomson-Haskell matrix method^54^, which allows for improving the numerical stability of the computation and accelerating the calculation of the dispersion curves. Figures S9 and S10 in the supplementary materials show some examples of the transdimensional inversion result, which consists of a probability distribution and the position of discontinuities for the S-wave velocity at each depth. The selected 1-D S-wave velocity model corresponds to the median probability value at each depth.

Figures S9A-B and C-D in the supplementary materials show the transdimensional inversion results corresponding to high-velocity anomalies H1 and H2, respectively. Figure S9A shows a rapid increase in S-wave velocity, starting from 1 km/s at the surface and reaching 4 km/s at 2 km depth. Three main discontinuities can be observed between the depths of 0 and 2 km (Fig. S9.B). Conversely, Figure S9.D shows a greater number of discontinuities in the superficial part above 1 km depth. At a depth of 1 km, the velocity begins to increase reaching approximately 3.0 km/s (Fig. S9.C).

Figure S10 shows four examples of transdimensional inversion results corresponding to low-velocity anomalies L1, L2, L3, and L4. Figures S10.A-B show the 1-D S-wave velocity profiles in depth for anomaly L1. A shallow discontinuity appears between 0 and 1 km depth, where the velocity increases rapidly, starting at 1 km/s and increasing until 2 km/s. Between 1 km and 3 km depth, the velocity is almost constant. At depths greater than 3 km, there is a velocity increase related to anomaly H2. Conversely, Figures S10.C-D show the 1-D S-wave velocity profiles in depth for anomaly L2. At shallow depths less than 1 km, there are multiple discontinuities with velocity increasing from 1 km/s to 2 km/s. At greater depths, the velocity remains almost constant. Figures S10.E-F and G-H show the 1-D S-wave velocity profiles in depth for anomalies L3 and L4, respectively. In both profiles, the velocity increases rapidly at shallow depths and does not vary significantly at greater depths.

In order to determine the depth resolution of our S-wave velocity model, we computed the group velocity sensitivity kernels for the fundamental mode of Rayleigh wave at different periods using the software senskernel-1.0^55^ (Fig. S11 of the supplementary materials). We note that the kernels have a sufficient resolution down to 3 km depth and no resolution below 5 km.

### Unification of S-wave velocity models

The S-wave velocity models obtained by ANT (this study) and LET^3^ have different resolutions as a function of depth. The ANT model has a higher resolution at a shallow depth of less than 4 km, while the LET model has a higher resolution at greater depths. The two models are therefore unified following the procedure described by D’Auria et al.^56^, where the two models are joined into a single one through a weighted averaging:

1 $$\begin{aligned} v(x_i, y_j, z_k) = \frac{\sum _{m=1}^{M} v_m(x_i, y_j, z_k) w_m (x_i, y_j, z_k)}{\sum _{m=1}^{M} w_m (x_i, y_j, z_k)} \end{aligned}$$

where $M$ is the number of models at the point $(x_i, y_j, z_k)$, $v_m(x_i, y_j, z_k)$ is the S-wave velocity and $w_m (x_i, y_j, z_k)$ is the weight of the m-th model. Figure S12 in the supplementary materials shows the weight as a function of depth for both S-wave velocity models. The S-wave velocity model unification was performed down to 5 km depth with weights varying between 0 and 1 (Fig. S12 in the supplementary materials). The unified S-wave velocity model has therefore a good resolution at both shallow and great depth, revealing the geometry of various low-velocity anomalies (Fig. S13 in the supplementary materials).

### Supplementary Information

Supplementary Information.

## Data Availability

All the results obtained in this study are shared at 10.5281/zenodo.7113144.
