# Supplementary material for: Geothermal and structural features of La Palma island (Canary Islands) imaged by ambient noise tomography
Source: Sci Rep. 2023 Aug 9;13:12892. doi: 10.1038/s41598-023-39910-z (PMC10412587; doi:10.1038/s41598-023-39910-z)
Supplement: Supplementary file 1 — Supplementary Information. [file 41598_2023_39910_MOESM1_ESM.pdf]

Supplementary materials for the article “Geothermal and structural features of La Palma island (Canary Islands) imaged by ambient noise tomography.”

Iván Cabrera-Pérez[1,*], Jean Soubestre[2, 3], Luca D’Auria[1,4], José Barrancos[4], Alba Martín-Lorenzo[4], David Martínez van Dorth[4], Germán D. Padilla[4], Monika Przeor[4] and Nemesio M. Pérez[1,4]

(1) Instituto Volcanológico de Canarias (INVOLCAN), 38600 Granadilla de Abona, Tenerife, Canary Islands, Spain.

(2) Univ. Grenoble Alpes, Univ. Savoie Mont Blanc, CNRS, IRD, Univ. Gustave Eiffel, ISTerre, 38000 Grenoble, France

(3) Icelandic Meteorological Office, Reykjavik, Iceland

(4) Instituto Tecnológico y de Energías Renovables (ITER), 38600 Granadilla de Abona, Tenerife, Canary Islands, Spain.

*corresponding: ivan.cabrera.perez1@gmail.com

List of Figures

Figure S1 shows the ray path density and anisotropy map.

Figure S2 shows a time-frequency representation of the dataset obtained by a network-based approach and used for the automatic detection and removal of time windows containing earthquakes.

Figure S3 shows the cross-correlations of all station pairs and the Rayleigh wave dispersion curves used in this study.

Figure S4 shows the checkerboard tests and the corresponding recovered group velocity maps.

Figure S5 shows some resolution tests on synthetic models composed of a pattern of low and high-velocity diamond-shaped anomalies, together with the corresponding recovered group velocity maps.

Figure S6 shows a resolution test on a synthetic model composed of anomalies similar to low-velocity anomalies L1 and L2 and high-velocity anomaly H1 imaged with real data, together with the corresponding recovered group velocity map.

Figure S7 shows the Rayleigh wave 2-D group velocity maps corresponding to a checkerboard test at different scales.

Figure S8 shows the Rayleigh wave 2-D group velocity maps corresponding to real data at different periods.

Figure S9 shows two examples of 1-D transdimensional inversion with relative posterior probability of discontinuities position corresponding to high-velocity anomalies H1 and H2.

Figure S10 shows four examples of 1-D transdimensional inversion with relative posterior probability of discontinuities position corresponding to low-velocity anomalies L1, L2, L3 and L4.
Figure S11 Group velocity sensitivity kernels for the fundamental mode of Rayleigh wave at different periods.
Figure S12 shows the weight function for unifying the S-wave velocity models obtained from ambient noise tomography (ANT) and local earthquake tomography (LET).
Figure S13 shows a comparison between the S-wave velocity model obtained from LET and the unified ANT+LET S-wave velocity model.

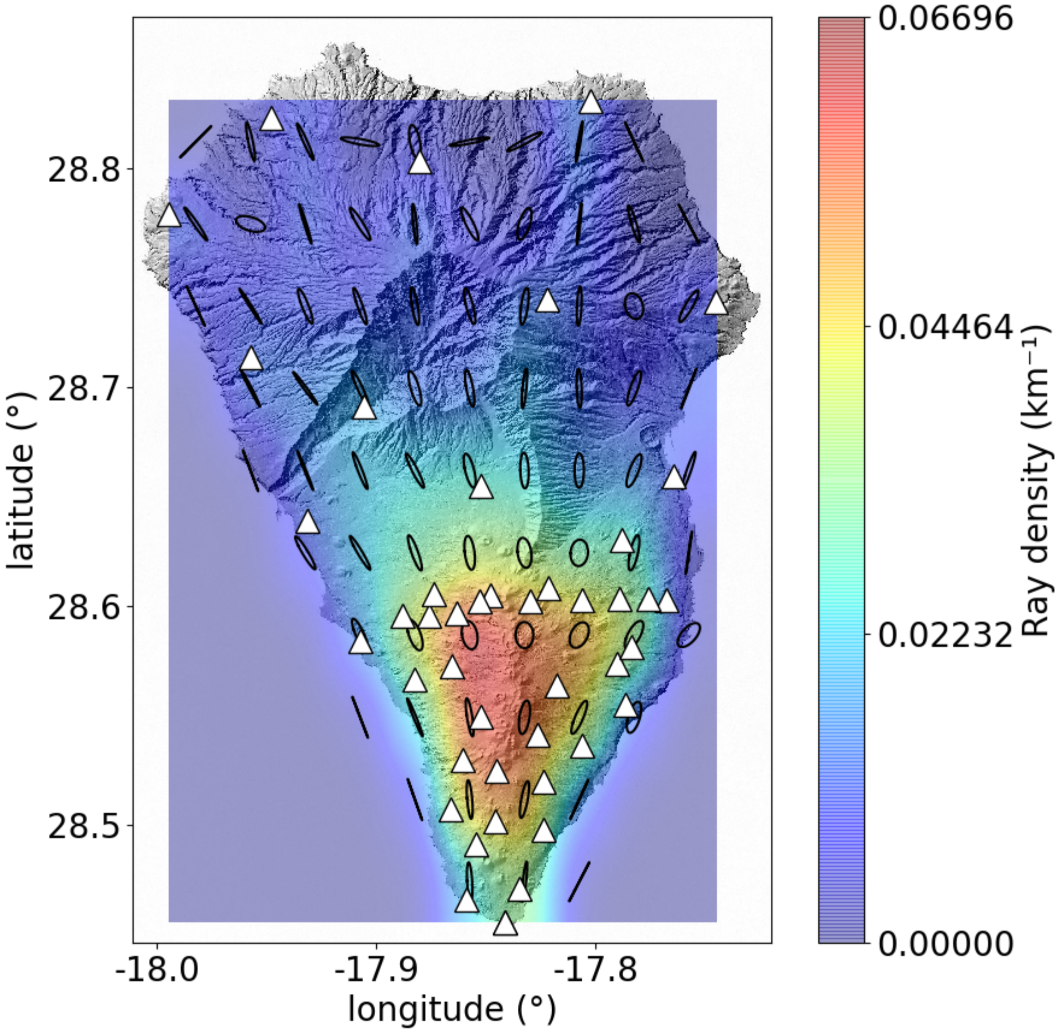

Figure S1. The map shows the distribution of seismic stations (white triangles) and the associated ray path density (colour bar). The ellipses represent the anisotropy of the ray paths.

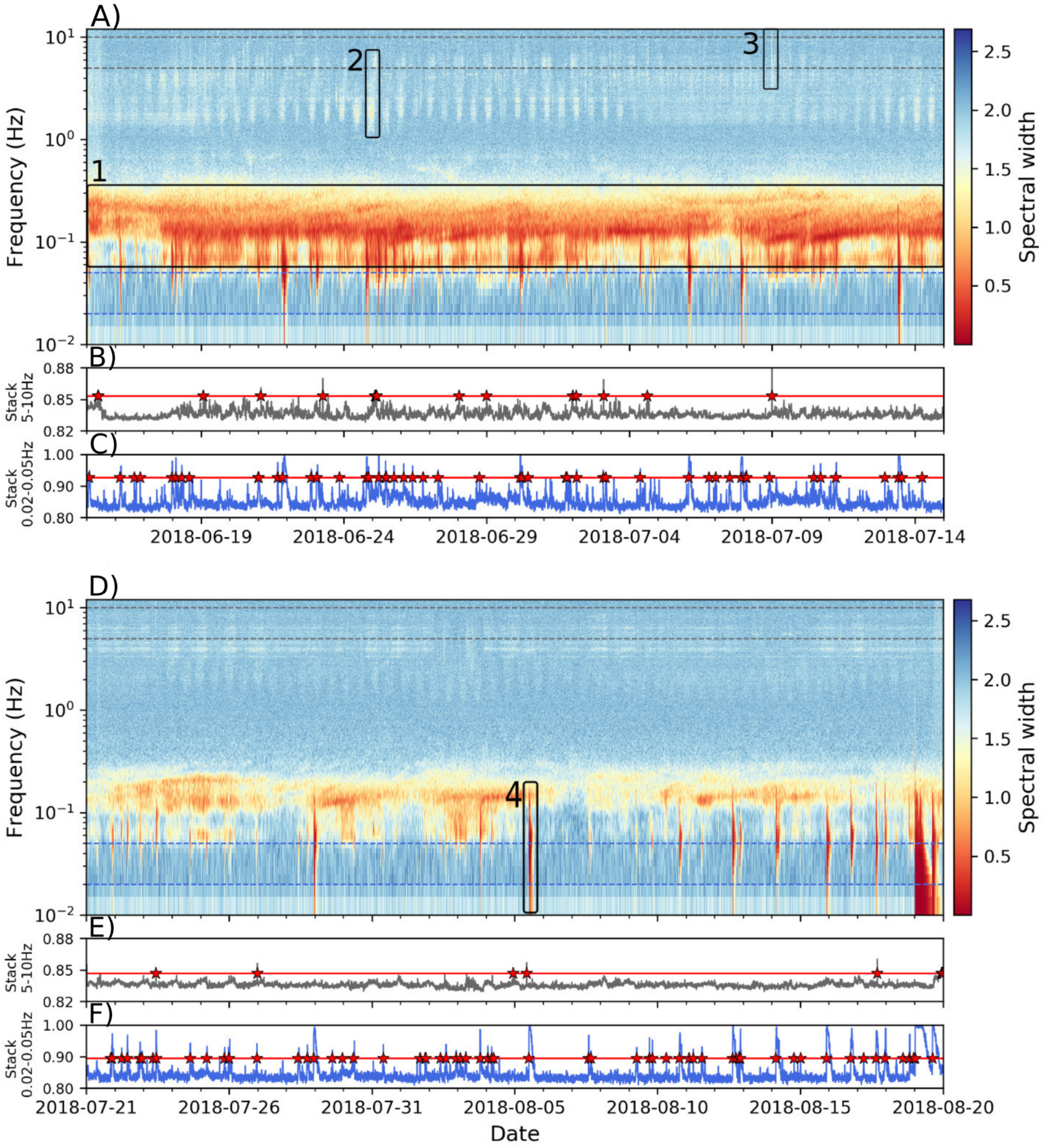

Figure S2. Panels A) and D) represent a time-frequency representation of the datasets during phases 1 and 2, respectively, obtained by a network-based approach. The stacks in frequency ranges between 5-10Hz and 0.02-0-05Hz appearing in panels B), C), E) and F) are used for the automatic detection and removal of time windows containing earthquakes. Squares 1 and 2 remark some oceanic and anthropic ambient noise, respectively. Squares 3 and 4 remark a volcano-tectonic and teleseismic earthquake, respectively.

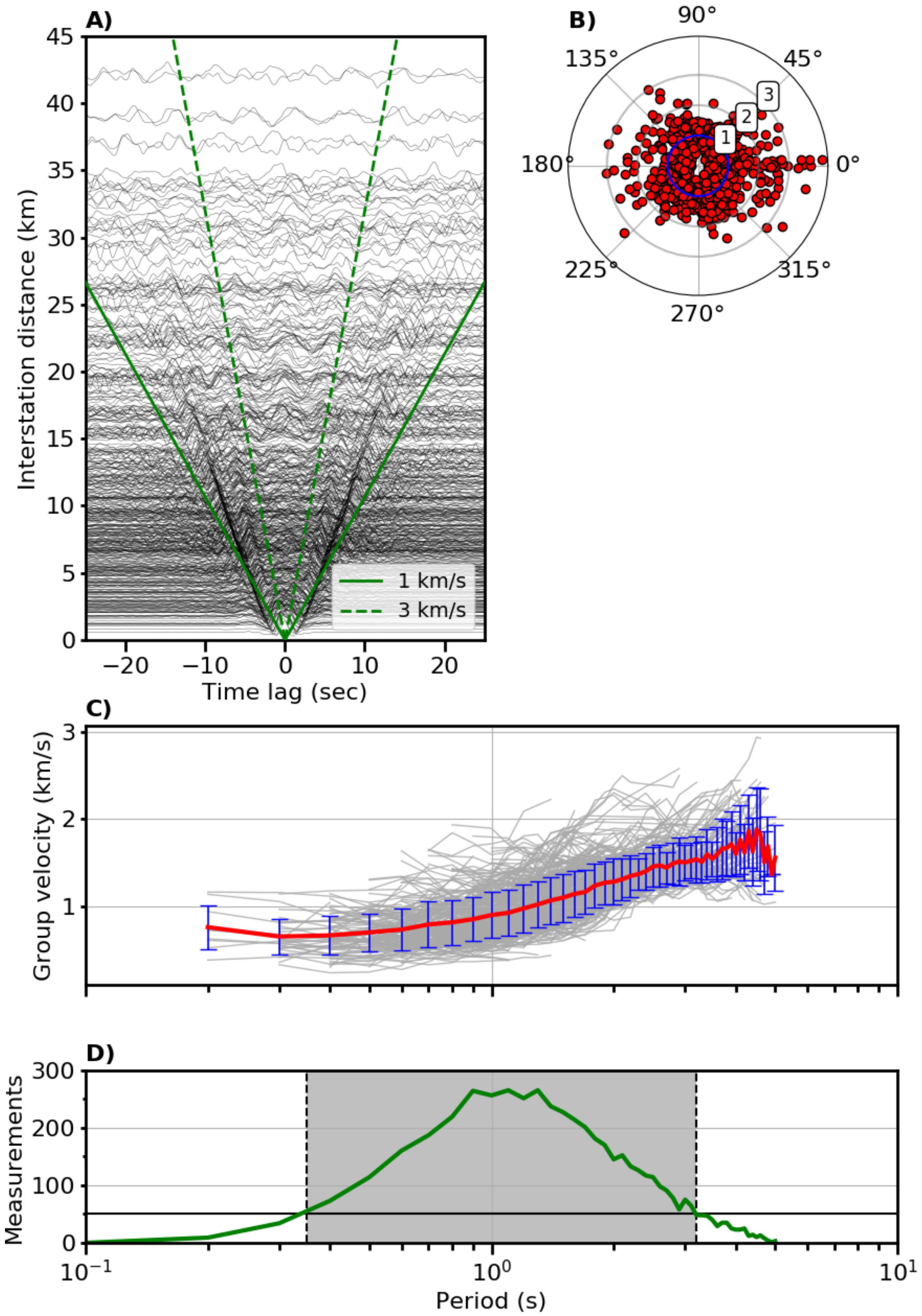

Figure S3. A) Cross-correlations of all station pairs are sorted according to the interstation distance. Velocities of 1.0 and 3.0 km/s are marked. B) Amplitude ratio of causal and acausal parts of cross-correlations as a function of the azimuthal distribution. The blue circle represents the unitary value of the amplitude ratio. C) Rayleigh wave dispersion curves (grey lines) used in this study. The average dispersion curve with the corresponding standard deviation (blue line) is in red. D) Number of measurements as a function of the period (green line). The grey square represents the period's range used in the inversion (0.35-3.2s).

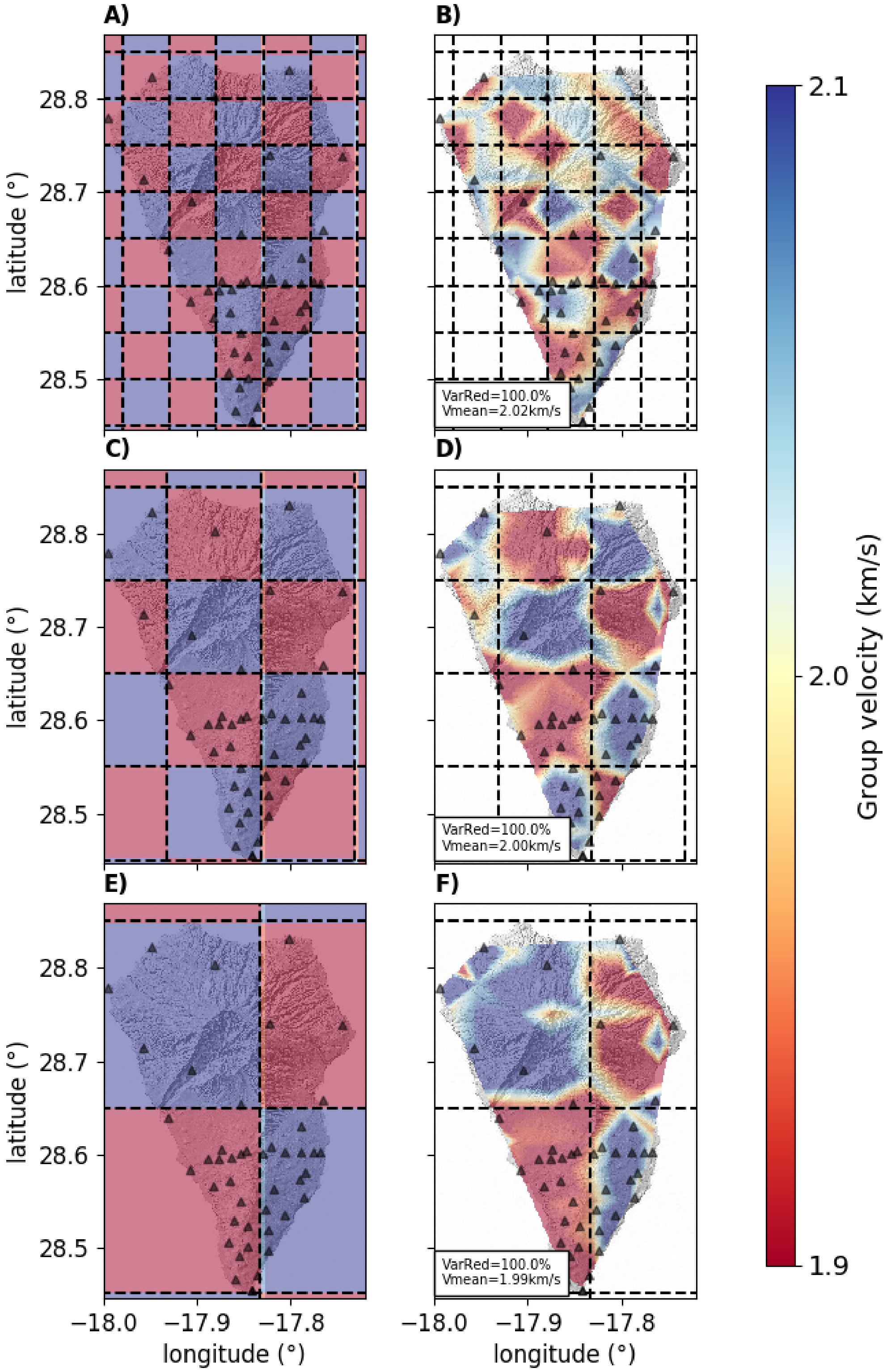

Figure S4. Checkerboard tests with size of (A) 0.05º x 0.05º, (C) 0.1º x 0.1º and (E) 0.2º x 0.2º. The corresponding recovered group velocity maps are shown in panels (B), (D) and (F), respectively.

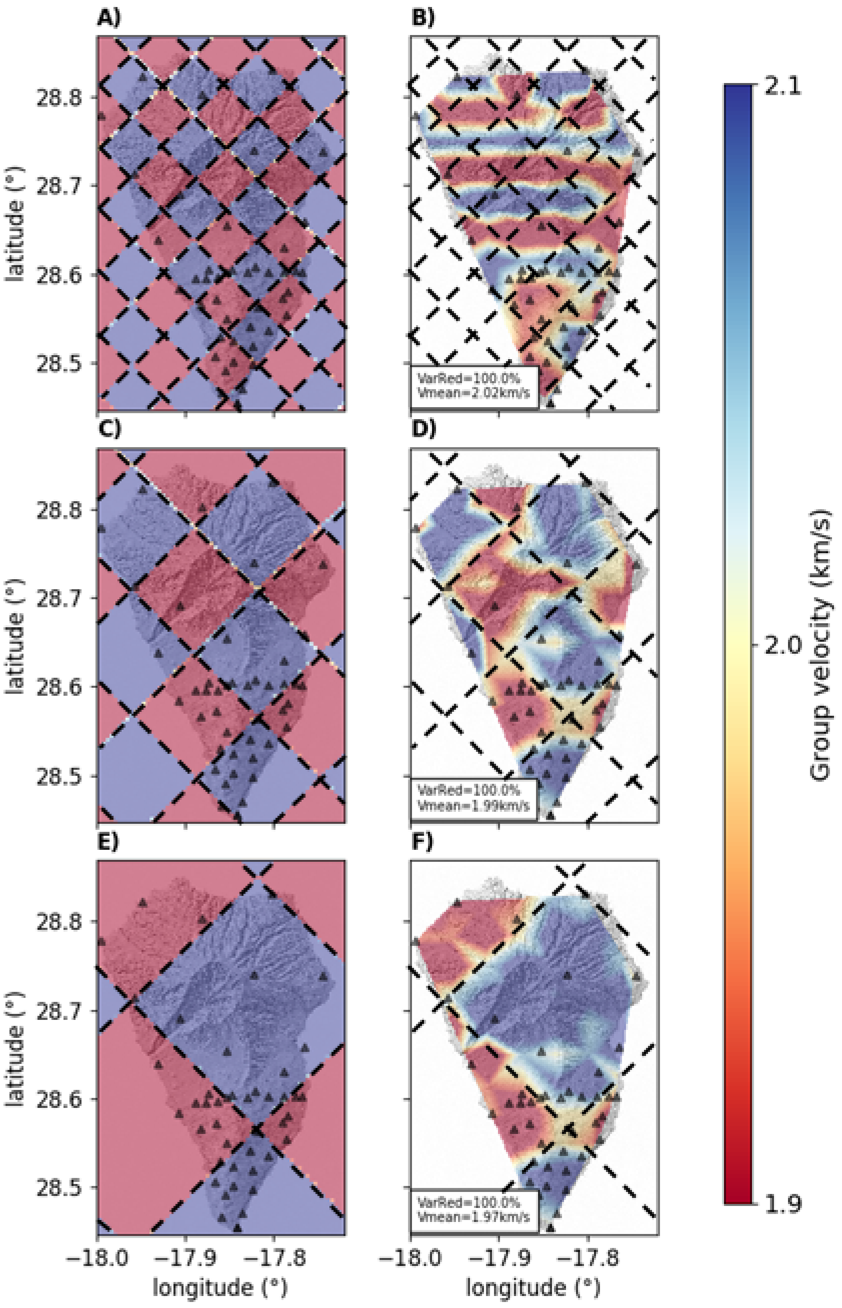

Figure S5. Resolution tests on synthetic models composed of a pattern of low and high-velocity diamond-shaped anomalies with size of(A) 0.05º x 0.05º, (C) 0.1º x 0.1º and (E) 0.2º x 0.2º. Panels (B), (D) and (F) show the corresponding recovered group velocity maps, respectively.

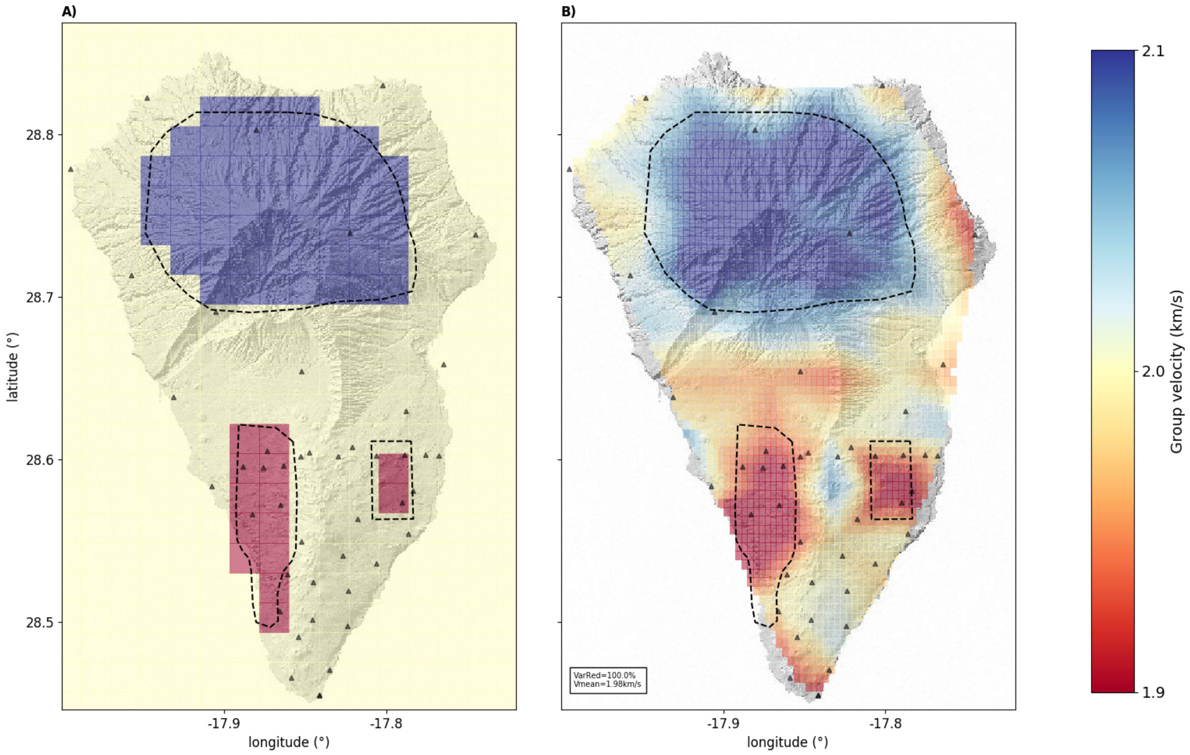

Figure S6. Resolution test on a synthetic model composed of anomalies similar to low-velocity anomalies L1 and L2 and high-velocity anomaly H1 imaged with real data (cf. Figure 2.C). Panel B) shows the retrieved model.

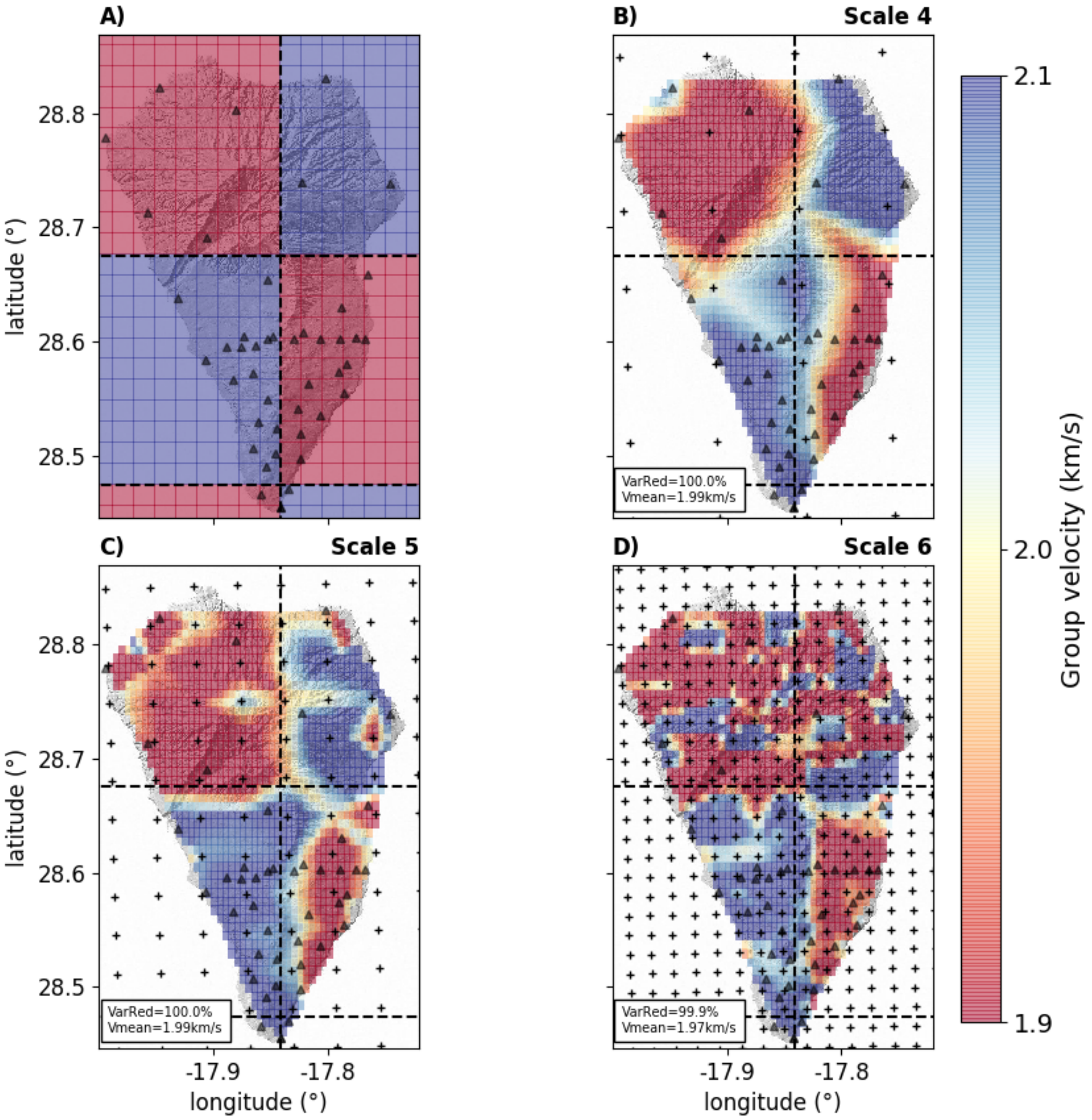

Figure S7. Rayleigh wave 2-D group velocity maps for different scales, B) scale 4, C) scale 5 and D) scale 6, obtained at a period of T=1.00 s for the checkerboard test represented in panel A). Black triangles and plus symbols represent the location of seismic stations and the nodes used in the inversion process, respectively. Variance reduction and mean velocity are indicated at the bottom-left corner of each panel.

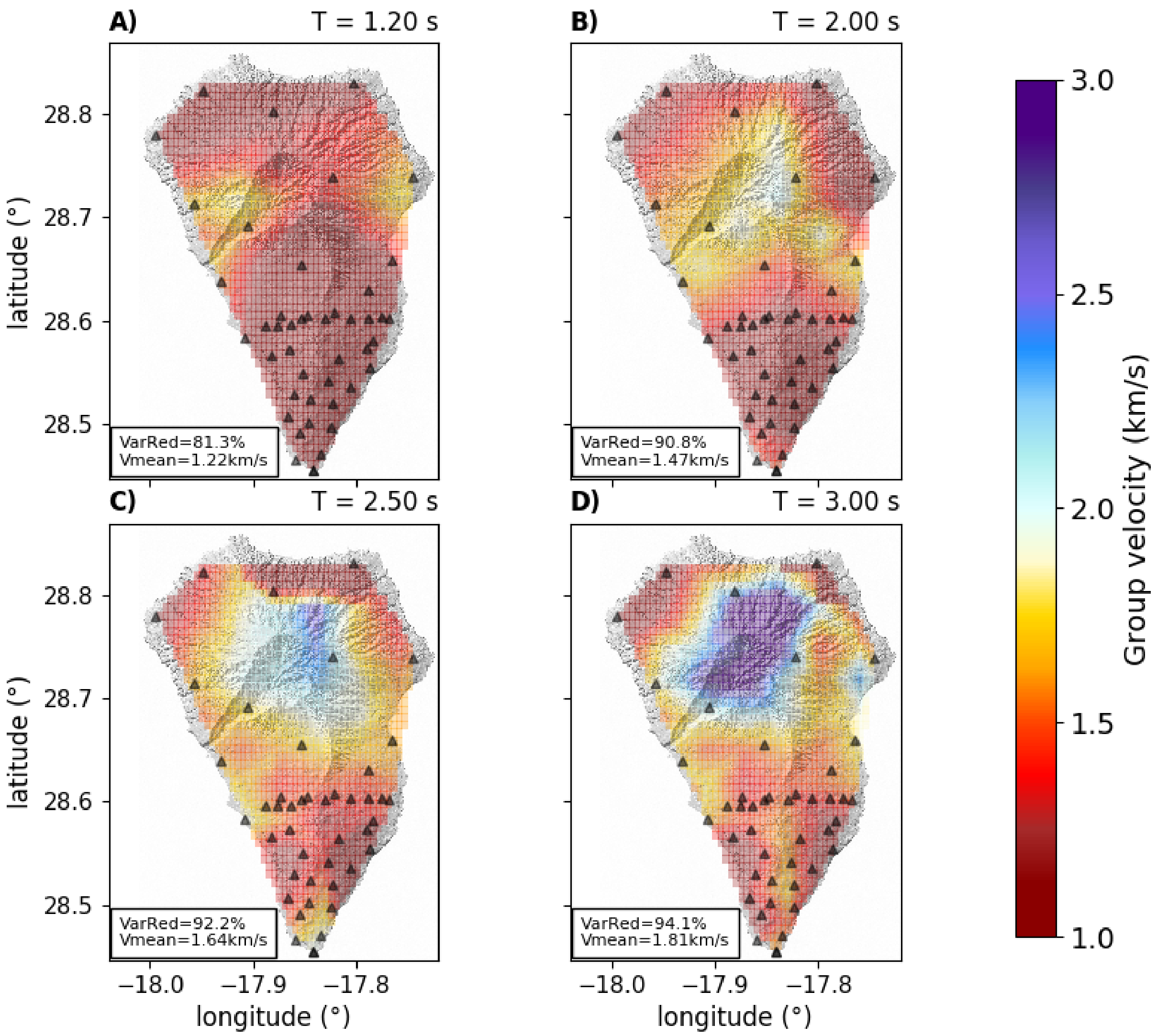

Figure S8. Rayleigh wave 2-D group velocity maps obtained from real data for different periods (indicated at the top-right of each panel) between T = 1.20 and T = 3.00 s. Black triangles represent the seismic stations. Variance reduction and mean velocity are indicated at the bottom-left corner of each panel.

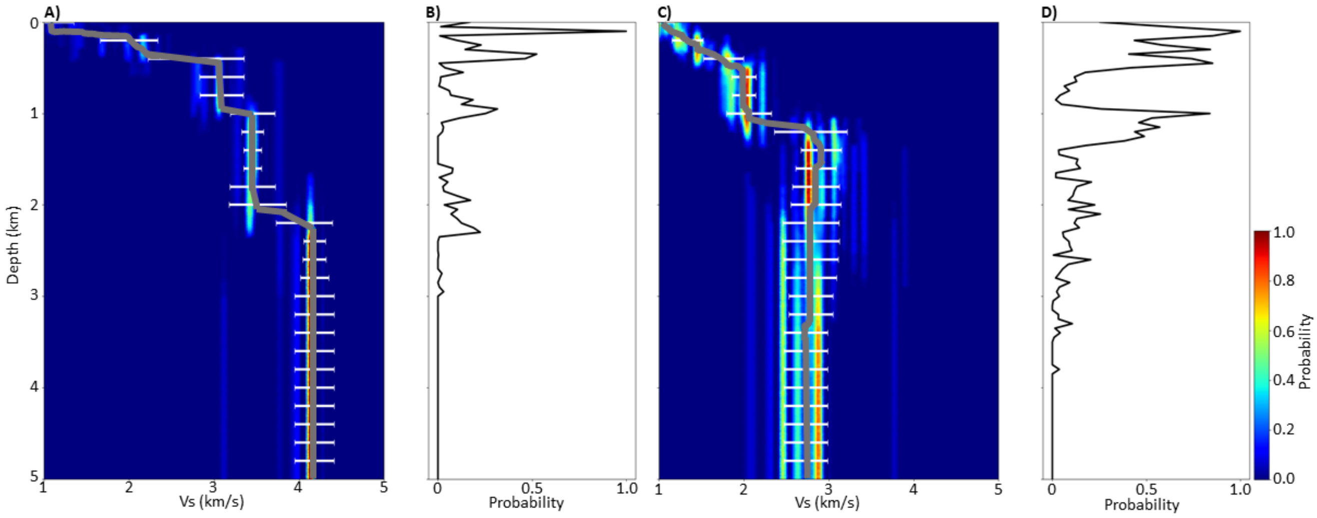

Figure S9. Examples of 1-D transdimensional inversion for high-velocity anomalies H1 (A and B) and H2 (C and D). The colour in panels A) and C) represents the probability distribution for Vs at each depth. The gray line and white bars indicate the median 1-D S-wave velocity model and the standard deviation at different depths, respectively. The extracted velocity model is represented by the gray line. Panels B) and D) represent the relative posterior probability for the position of discontinuities at each depth.

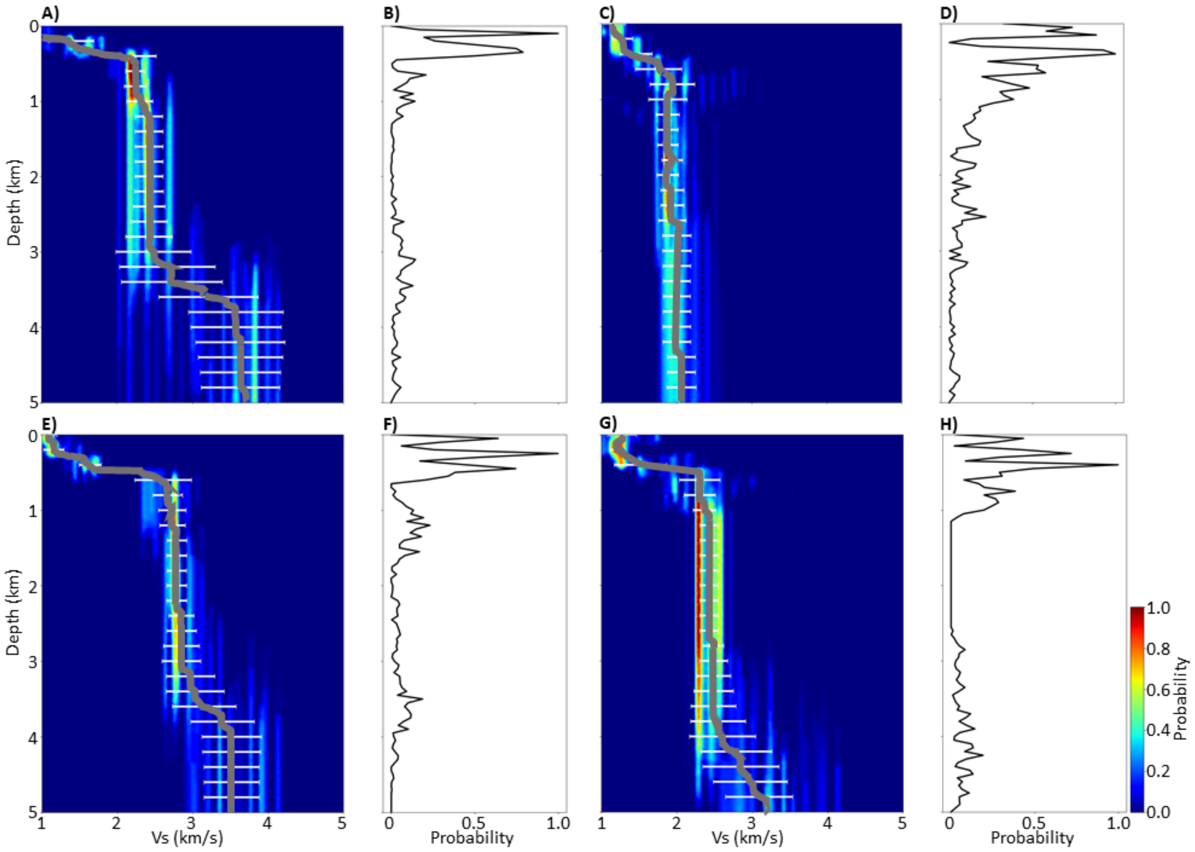

Figure S10. Examples of 1-D transdimensional inversion for low-velocity anomalies L1 (A and B), L2 (C and D), L3 (E and F) and L4 (G and H). The colour in panels A), C), E) and G) represents the probability distribution for Vs at each depth. The gray line and white bars indicate the median 1-D S-wave velocity model and the standard deviation at different depths, respectively. The extracted velocity model is represented by the gray line. Panels B), D), F) and H) represent the relative posterior probability for the position of discontinuities at each depth.

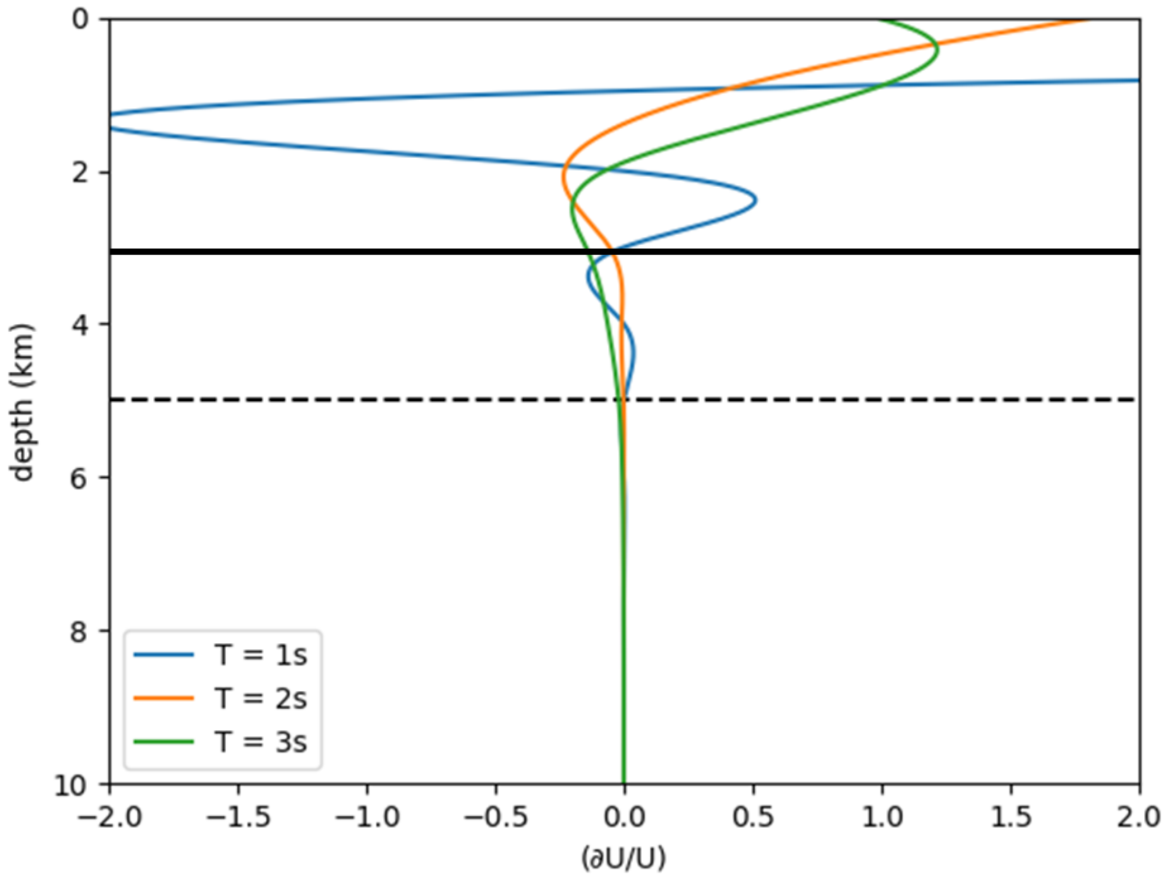

Figure S11. Group velocity sensitivity kernels for the fundamental mode of Rayleigh wave at T = 1 s (blue line), T = 2 s (orange line), and T = 3 s (green line) periods. Kernel is based on the 1-D mean S-wave velocity model. The horizontal black solid and dashed lines denote the maximum resolution and the limit of resolution at depth of our model, respectively.

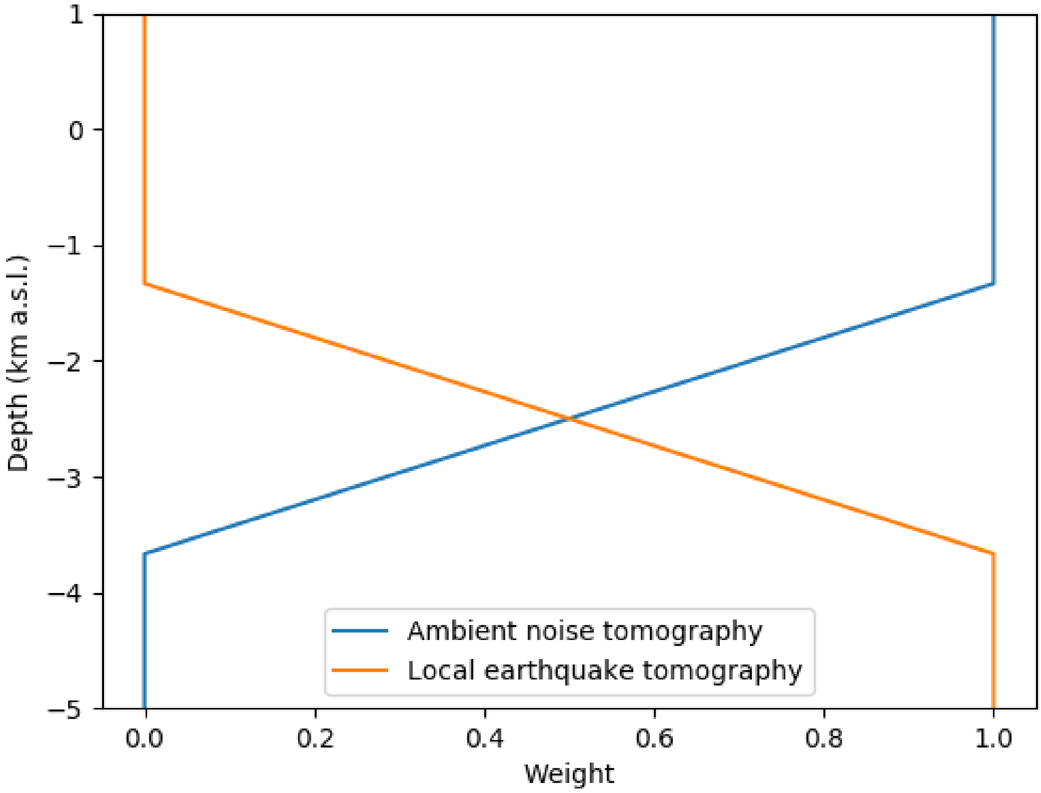

Figure S12. Weight function for the unification of the S-wave velocity models. The blue and orange lines represent the weight as a function of depth for the velocity models obtained from ambient noise tomography (ANT) and local earthquake tomography (LET), respectively.

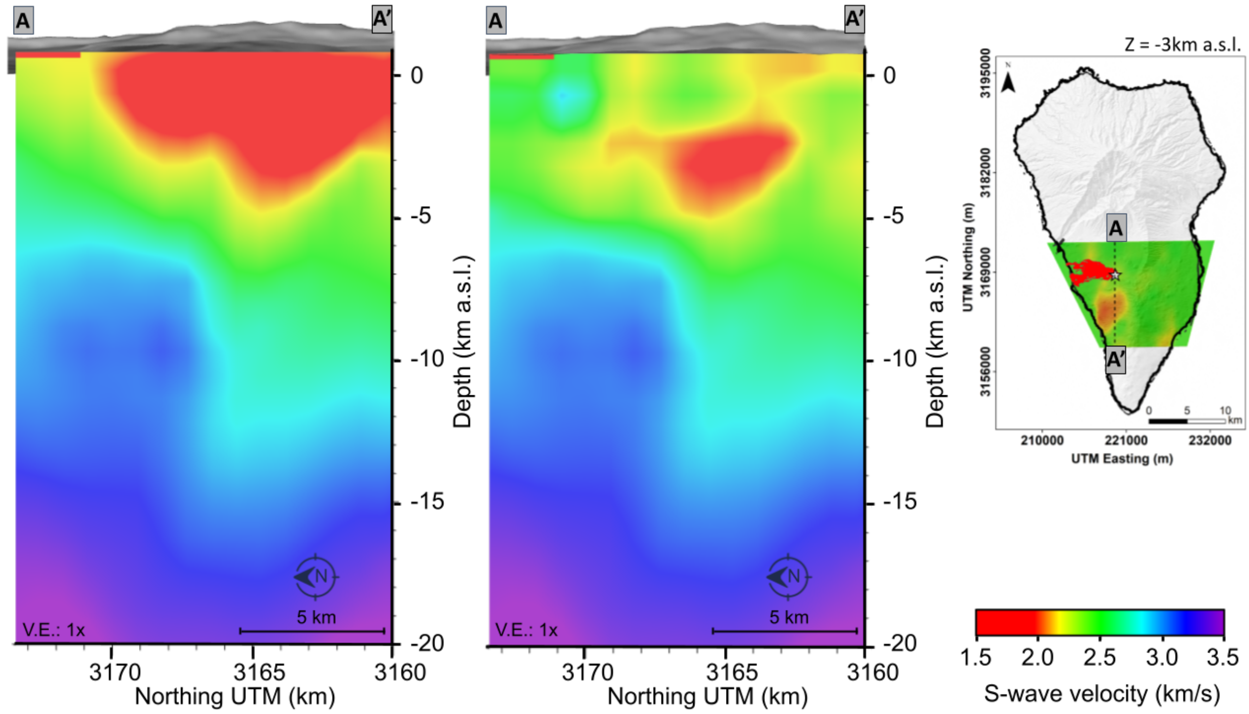

Figure S13. Vertical cross-section of the S-wave velocity model obtained from LET (left panel) and of the unified ANT+LET S-wave velocity model (center panel). The map on the right panel shows a horizontal cross-section of the unified ANT+LET velocity model at -3000 m.a.s.l.. The white star and red contour represent the location of the eruptive event and lava flows.
